# MiRNAs and E2F3: a complex network of reciprocal regulations in human cancers

**DOI:** 10.18632/oncotarget.17364

**Published:** 2017-04-21

**Authors:** Yanping Gao, Bing Feng, Lu Lu, Siqi Han, Xiaoyuan Chu, Longbang Chen, Rui Wang

**Affiliations:** ^1^ Department of Medical Oncology, Jinling Hospital, School of Medicine, Nanjing University, Nanjing, Jiangsu, PR China

**Keywords:** microRNA, E2F3, proliferation, apoptosis, metastasis

## Abstract

E2F transcription factor 3 (E2F3) is oncogenic in tumorigenesis. Alterations in E2F3 functions correspond with poor prognosis in various cancers, underscoring their status for the clinical cancer phenotype. Latest reports discovered intricate networks between microRNAs (miRNAs) and E2F3 in regulating the balance of these events, including proliferation, apoptosis, metastasis, as well as drug resistance. miRNAs are non-coding small RNAs which negatively regulate gene expressions post-transcriptionally mainly through 3′-UTR binding of target mRNAs. Increasing evidence shows that E2F3 can be activated/inhibited by numerous miRNAs whose dysregulation has been implicated in malignancy. In turn, miRNAs themselves can be transcriptionally regulated by E2F3, thus forming a negative feedback loop. These findings add a new challenging layer of complexity to E2F3 network. Current understanding of the reciprocal link between E2F3 and miRNAs in human cancers were summarized, which could help to develop potential therapeutic strategies.

## INTRODUCTION

E2F transcription factor 3 (E2F3) belongs to E2F family and has two distinct isoforms, E2F3a and E2F3b, functioning through binding of DP interaction partner proteins [1]. E2F3 locates in Chromosome 6, NC_000006.12. To data, the E2F family is reported to be composed of members, such as E2F1, E2F2, E2F3a, E2F3b and E2F4-E2F8. Meanwhile, the transcriptional activators E2Fs play vital roles in regulation of cell cycle and governing cell proliferation by releasing from pRb and binding to the promoters of target genes, but repressor E2Fs often have weak activation potential [2, 3]. E2F acts as a crucial regulator of the cell cycle, and its dysregulation contributes to oncogenesis. The E2F family members can integrate their different signalings and the complex crosstalks between them facilitate ordered or disordered progression for the determination of cell fate in various human tissues. The DNA binding activity of E2F3 shows a significant decrease when cells progress through S phase. During G0-phase of cell cycle, E2F3b can be observed to be connected with E2F-binding elements on promoters of E2F-target genes. E2F3a does not show up in quiescent cells, it only accumulates when cells enter G1 and helps to carry out the cell cycle [4]. In some situation, it is important for E2F3 to regulate cellular proliferation of both primary and tumor cells *via* induction of apoptosis by DNA damage [5, 6]. Meanwhile, mutation of E2F3 led to partial embryonic lethality, which could be amplified by mutated E2F1 or E2F2 [7]. Altered copy number of E2F3 and changes in its transcriptional activity have been found in a variety of human malignancies. Recent studies have discovered several pathways that directly regulate multifunctional E2F3, presenting a significant tumor supervisor gene and a promising therapeutic target for the treatment of human cancers.

It has been generally accepted that miRNAs are a group of 19∼24 nucleotide-sized small non-coding RNAs functioning in target gene regulation at the transcriptional or post-transcriptional level [8, 9]. Currently, miRNAs have been considered to be components of multi-level regulatory networks vast regulatory networks in cells. Several findings have shown that miRNAs can function as oncogenes or tumor suppressors by regulating different target genes in various human cancers, so the field is focused on identification of direct and functional target genes of individual miRNAs [10]. With more in-depth understanding of miRNAs, they have been considered as novel biomarkers for cancer diagnosis and prognosis and promising tools for cancer treatment.

A single miRNA can regulate multiple target mRNAs, while invidual mRNAs can be targeted by several miRNAs [11]. Considering this special peculiarity, it is not surprising that miRNAs can be directly or indirectly restricted by their target genes. Current findings indicated that these bi-directional circuits exist widely in different miRNAs and their targets. Then, the regulatory feedback loops have been clarified the molecular networks one after another, such as ZEB/miR-200, Notch/miR-326, E2F1/miRNA-223, NF-κB/miR-200b, c-Myb/miR-15a and p53/ miR-200 [12–17]. From here we see that the individual targeting of one miRNAs to its seed-matched mRNA may be much weaker, but the combination of multiple miRNAs targeting genes that match in the same 3′-UTR can exert big biological effects [18]. Identification of the reciprocal link between E2F3 and miRNAs may help to extended our understanding of carcinogenesis and develop potential therapeutic strategies against cancer.

## MIRNA-E2F3 INTERACTIVE RELATION: MIRNAS REGULATE E2F3

### Transcriptional factors

**Figure 1:**
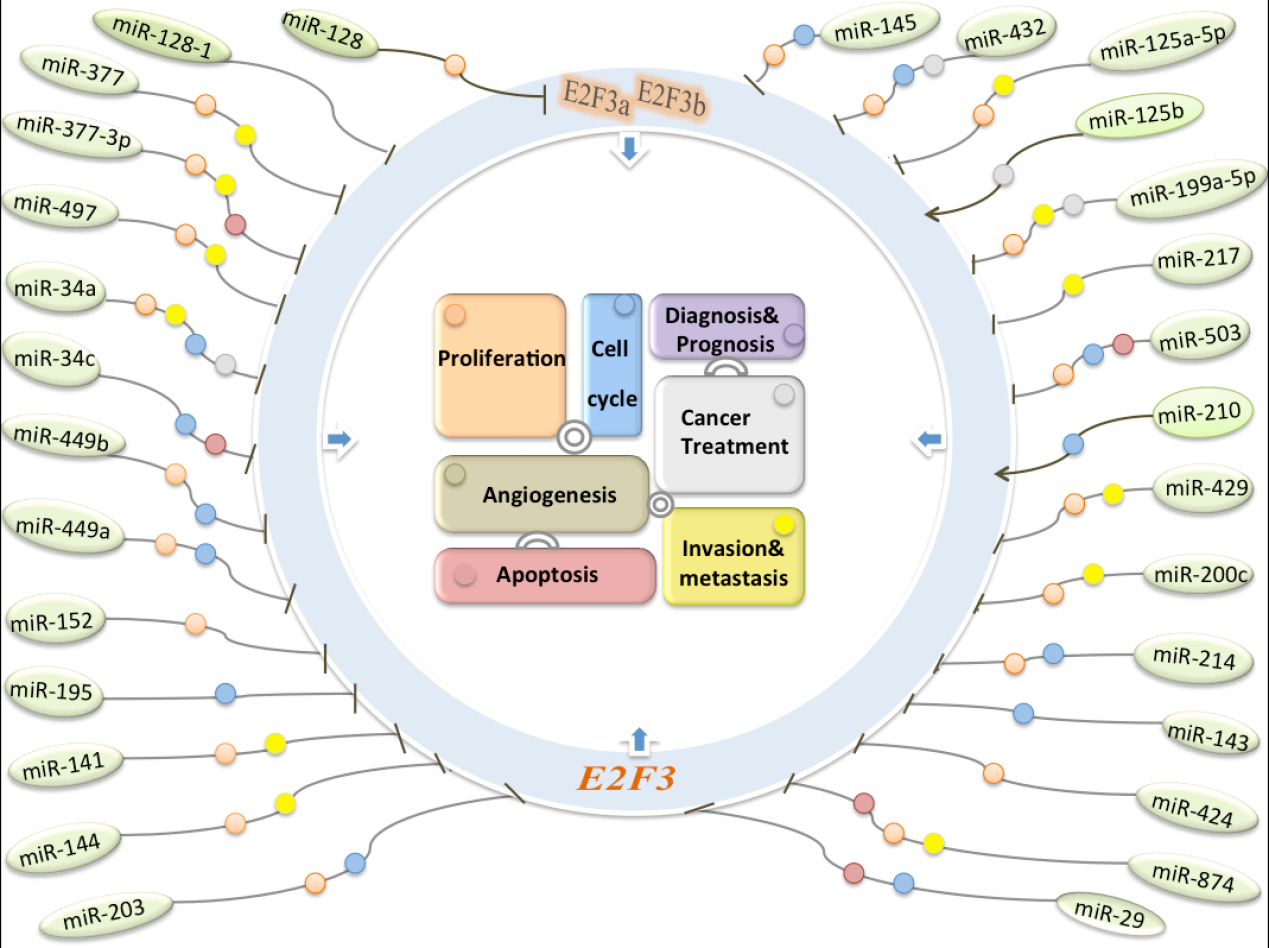
The molecular regulatory networks about miRNAs regulate E2F3 transcription factors in human cancers The miRNA-E2F3 interactive relation play an intricate role in tumorigenicity including cell proliferation, apoptosis, cell cycle, metastasis, and multiple clinical treatment steps (different round colors).

The multiple relationships between miRNA or E2F3 and human cancers, as well as the finding that E2F3 is coordinated by miRNAs have attracted more attention from numerous researchers. Here's the section that summarizes the better-characterized contacts between tumor-related miRNAs and E2F3 (Table 1a, 1b, 1c, 1d; Figure 1).

**Table 1a:** miRNAs directly target E2F3 in human cancers

miRNAs	Cancer types	miRNA alterations	Mechanism of action and validation	Cell functions	Target genes of miRNA	References
miR-128	glioma	Down regulated	Targets 3′-UTR of E2F3a (luciferase assay)	Inhibits proliferation	--	[19]
	GBM	Down regulated	Targets 3′-UTR of E2F3a (luciferase assay)	Inhibits proliferation	--	[20]
miR-128-1	GBM	Down regulated	Targets 3′-UTR of E2F3 (luciferase assay)	Inhibits growth	--	[21]
miR-377	Melanoma	Down regulated	Targets 3′-UTR of E2F3 (luciferase assay)	Inhibits proliferation, migration	--	[22]
miR-377-3p	NSCLC	Down regulated	Targets 3′-UTR of E2F3 (luciferase assay)	Inhibits proliferation, migration; Promotes apoptosis	--	[23]
miR-34a	AML	Down regulated	Targets 3′-UTR of E2F3 (luciferase assay)	Blocks the transition from the G0/G1 to S phase of cell-cycle progression	--	[24]
	Ovarian cancer	Down regulated	Targets 3′-UTR of E2F3a and E2F3b (luciferase assay)	Affects cell-cycle progression and cell proliferation	--	[25]
	NB	Down regulated	Targets 3′-UTR of E2F3	Antiproliferative	--	[26] [27]
	Colon cancer	Down regulated	Targets 3′-UTR of E2F3 (luciferase assay)	Proliferation negatively regulate the resistance to 5-FU in colorectal cancer DLD-1 cells	--	[28]

**Table 1b:** miRNAs directly target E2F3 in human cancers

miRNAs	Cancer types	miRNA alterations	Mechanism of action and validation	Cell functions	Target genes of miRNA	References
miR-34a	Colorectal cancer	Down regulated	Targets 3′-UTR of E2F3 (luciferase assay)	Results in accumulation of dUTP and reduced dTTP biosynthesis, potentially enhancing 5-FU cytotoxicity	E2F1	[29]
	Breast cancer	Down regulated	Targets 3′-UTR of E2F3 (luciferase assay)	Suppresses cell growth, migration, and invasion	--	[30]
	HNSCC	Down regulated	Targets 3′-UTR of E2F3		Surviving VEGF	[31]
	Cervical cancer	Down regulated	Targets 3′-UTR of E2F3 (luciferase assay)	Reduces the viability and invasion	survivin	[35]
miR-34c	EC	Down regulated	TargetScan analysis; Western blot analysis	Inhibits cell proliferation, migration and invasion; Induces cell cycle arrest and apoptosis	--	[36]
miR-152	EC	Down regulated	Targets 3′-UTR of E2F3 (luciferase assay)	Targets the DNA methyltransferase; Inhibits tumor cell growth	--	[37]
miR-195	Glioblastoma	Down regulated	Targets 3′-UTR of E2F3 (luciferase assay)	Arrests cell cycle progression	--	[39]
miR-497	BTCC	Down regulated	Targets 3′-UTR of E2F3 (luciferase assay)	Inhibits the proliferation, migration and invasion	--	[40]
miR-203	Melanoma	Down regulated	Targets 3′-UTR of E2F3 (luciferase assay)	Inhibits cell growth; Induces senescence by cell cycle arrest	--	[41]
	Glioma	Down regulated	Targets 3′-UTR of E2F3 (luciferase assay)	Inhibits migration and invasion	--	[42]
	NPC	Down regulated	Targets 3′-UTR of E2F3 (luciferase assay)	Inhibites EBV-induced S-phase entry and transformation in vivo	--	[43]

**Table 1c:** miRNAs directly target E2F3 in human cancers

miRNAs	Cancer types	miRNA alterations	Mechanism of action and validation	Cell functions	Target genes of miRNA	References
miR-449b	Colorectal cancer	Down regulated	Targets 3′-UTR of E2F3 (luciferase assay)	Inhibits proliferation; Regulars cell cycle	--	[44]
miR-449a	Lung cancer	Down regulated	Targets 3′-UTR of E2F3 (luciferase assay)	Causes cell cycle arrest and cell senescence	--	[45]
	Gastric cancer	Down regulated	Targets 3′-UTR of E2F3 (luciferase assay)	Inhibits Proliferation; Induces Apoptosis	--	[46]
miR-503	HCC	Down regulated	Targets 3′-UTR of E2F3 (luciferase assay)	Suppresses proliferation and induces of G1 phase arrest	--	[48]
	CRC	Down regulated	Targets 3′-UTR of E2F3 (luciferase assay)	Inhibits proliferation, induces apoptosis and G0/G1 arrest	--	[49]
miR-144	HCC	Down regulated	Targets 3′-UTR of E2F3 (luciferase assay)	Suppresses the proliferation and metastasis	--	[50]
miR-217	HCC	Down regulated	Targets 3′-UTR of E2F3 (luciferase assay)	Suppresses invasion	--	[51]
miR-424	HCC	Down regulated	Targets 3′-UTR of E2F3 (luciferase assay)	Suppresses the tumor growth	--	[52]
miR-214	HCC	Down regulated	Targets 3′UTR of E2F3 (luciferase assay)	Induces G1-S phase arrest and inhibits cell proliferation	--	[83]
miR-199a-5p	HCC	Down regulated	Targets 3′-UTR of E2F3 (luciferase assay)	Inhibits tumor growth and migration/invasion; Sensitizes mesenchymal liver tumor cells to chemotherapeutics	--	[53]

**Table 1d:** miRNAs directly target E2F3 in human cancers

miRNAs	Cancer types	miRNA alterations	Mechanism of action and validation	Cell functions	Target genes of miRNA	References
miR-143 /miR-145	Colon cancer	Down regulated	Only by Western blot	Restrains role on cell cycle	--	[54]
miR-210	Pancreatic cancer	Up regulated	Targets 3′-UTR of E2F3 (luciferase assay)	MiR-210 induced by hypoxia through a HIF-1α-dependent	--	[56]
	Ovarian cancer	Up regulated	Only by Western blot	Links hypoxia with the regulation of cell cycle	--	[57]
	Lung adenocarcinoma	Up regulated	Only by qRT-PCR	Induces pro-tumourigenic	--	[59]
miR-432	LAD	Down regulated	Targets 3′-UTR of E2F3 (luciferase assay)	Inhibits cell proliferation through arresting cell cycle and sensitizes tumor cells to cisplatin	--	[60]
miR-125a-5p	Gastric cancer	Down regulated	Targets 3′-UTR of E2F3 (luciferase assay)	Inhibites proliferation, migration and invasion	--	[62]
miR-125b	Breast cancer	Up regulated	Targets 3′UTR of E2F3 (luciferase assay)	Associates with chemotherapeutic resistance	--	[63]
	Prostate cancer	Up regulated	By Western blot and qRT-PCR	Contributes to PCa aggressiveness	--	[64]
miR-145	Gastric cancer	Down regulated	Targets 3′-UTR of E2F3 (luciferase assay)	Inhibites proliferation, cell viability and induced cell arrest in S-phase	--	[47]
miR-29	Osteosarcoma	Down regulated	Only by Western blot	Induces apoptosis and cell cycle arrest	--	[65]
miR-874	Osteosarcoma	Down regulated	Targets 3′-UTR of E2F3 (luciferase assay)	Inhibits proliferation, migration, and invasion and induce cell apoptosis	--	[66]
miR-200c	Bladder cancer	Down regulated	Targets 3′-UTR of E2F3 (luciferase assay)	Inhibites proliferation, migration and invasion; Affects EMT	--	[67]
miR-141	HCC	Down regulated	Targets 3′-UTR of E2F3 (luciferase assay)	Suppresses the proliferation, migration and invasion	--	[68]
miR-429	RCC	Down regulated	Targets 3′-UTR of E2F3 (luciferase assay)	Suppresses cell proliferation, epithelial-mesenchymal transition and metastasis	--	[69]

Previously, it was reported that miR-128 was down-regulated and E2F3a was highly expressed in glioma cells or tissues, and upregulation of miR-128 significantly reduced the luciferase activity of a luciferase-reporter containing the 3′-UTR of E2F3a mRNA [19]. Conformably, miR-128 was also observed to be significantly reduced in glioblastoma multiforme (GBM) tissues and cell lines and E2F3a was its bioinformatics-verified target [20]. A recent study suggested that miR-128-1 was downregulated in GBM and glioma stem-like cells (GSCs) and miR-128-1 could inhibit the growth of glioblastoma multiforme and glioma stem-like cells *via* targeting BMI1 and E2F3 [21]. Scholars hold the view that miR-377 regulates the NF-κB signaling pathway through MAP3K7 by directly targeting E2F3 in malignant melanoma. Importantly, there was almost no expression of miR-377 in melanoma cells or tissues [22]. A recent study indicated that long non-coding RNA (lncRNA)-nuclear enriched abundant transcript 1 (NEAT1) acted as a ceRNA for miR-377-3p and thereby modulated the derepression of its endogenous target E2F3 in NSCLC [23].

The miR-34 family consists of miR-34a, 34b and 34c, which are dysregulated in various human cancers. Pulikkan JA and his colleagues reported that miR-34a was downregulated and E2F3 was elevated in AML samples with CEBPA mutations and C/EBPα-regulated miRNA-34a targeted E2F3 during granulopoiesis in AML [24]. In ovarian cancer, both E2F3a and E2F3b were over-expressed as compared with normal ovarian tissue, but E2F3a expression by far exceeded than E2F3b. Meanwhile, two EGFR-independent mechanisms caused by methylation of miR-34a were further identified to be involved in regulation of E2F3a expression. Also, it was found that miR-34a could physically interact with E2F3a transcripts and cause E2F3a degradation [25]. Follow-up studies suggested that miR-34a could function as a tumor suppressor in Neuroblastoma (NB) tumorgenesis by directly binding to E2F3 mRNA [26, 27]. Also, miR-34a in 5-FU-resistant colorectal cancer (CRC) cells was found to be significantly under-expressed compared with the parental cells after 5-FU treatment and restoration of miR-34a reversed the 5-FU resistance by down-regulation of Sirt1 and E2F3 [28]. Kiyonari et al. found that p53-dependent repression of nuclear isoform of dUTPase (DUT-N) expression by oxaliplatin was preceded by induction of pri-miR-34a expression and repression of expression of the transcription factors E2F3 and E2F1 in CRC [29]. In breast cancer, miR-34a was also found to be down-regulated, and restoration of miR-34a showed obvious anti-tumor effects *in vitro* and *in vivo* by targeting E2F3 [30]. Several other studies demonstrated that overexpression of miR-34a markedly attenuated E2F3 and survivin levels in head and neck squamous cancer (HNSC) cells [31]. Survivin, which belongs to the inhibitor of apoptosis proteins (IAP) family, plays a critical role in tumor progression and chemo- or radioresistance and its promoter activity was reported to be regulated by E2F3 [32–34]. Upregulation of E2F3a could completely rescue the expression of survivin protein in miR-34a-expressed HNSC cells, suggesting that miR-34a may reduce the expression of survivin through E2F3a. Also, miR-34a down-regulation was detected in cervical cancer, while the expression of miR-34a showed a decreasing trend from normal HR-HPVY negative tissue, normal HR-HPVY positive tissue, and HR-HPVY positive CIN cases to cervical cancer. In addition, miR-34a was proved to suppress survivin expression in cervical cancer by targeting E2F3, confirming the existence of novel miR-34a-E2F3a-survivin axis [35]. In endometrial carcinoma (EC), miR-34c was significantly reduced and E2F3 was reduced after up-regulation of miR-34c in the HEC-1-B cell, suggesting that miR-34c functions *via* reduction of E2F3 protein [36]. Another study showed that miR-152 functions as a tumor suppressor in EC, and E2F3, MET, and Rictor were identified as novel candidate targets of miR-152 [37].

The miR-15 family members, including miR-15a, miR-15b, miR-16-1, miR-16-2, miR-195 and miR-497, are clustered on three separate chromosomes [38]. In human glioblastoma cells, miR-195 was testified to function as a tumor suppressor by targeting E2F3 [39]. In another study, miR-497 was found to be markedly down-regulated in human bladder transitional cell carcinoma (BTCC) tissue samples and dual-luciferase reporter assays demonstrated the specificity of miR-497 to target E2F3 [40]. Meanwhile, E2F3a and E2F3b were testified as targets of miR-203 in human malignant melanoma Mewo cells [41]. Another study showed that miR-203 expression was also lower in highly invasive glioma cells or tissues and the inverse expression patterns between miR-203 and E2F3 in invasive glioma tissues were verified [42]. E2F3 and CCNG1 was identified as targets of miR-203 in nasopharyngeal carcinoma (NPC), while latent membrane protein 1 (LMP1) was responsible for downregulation of miR-203 [43].

The 3′-UTRs of E2F3 contained miR-449b binding sites, while E2F3 was also reported to be a direct target of miR-449a [44, 45]. It was also reported that miR-449a and miR-145 played tumor suppressive roles by targeting E2F3 in gastric cancer [46, 47]. On chromosomal location Xq26.3, miR-503 was an intragenic miRNA clustered with miR-424, which was significantly decreased in HCC and colorectal cancer (CRC) and functioned as a tumor suppressor by directly targeting E2F3 [48, 49]. Four miRNAs (miR-144, miR-217, miR-424 and miR-214) were also reported to be down-regulated in HCC, and the supplement of their target gene E2F3a partially reversed the tumor suppressive effects of these miRNAs in HCC cells [50–52]. In HCC, miR-199a-5p levels were down-regulated accompanied by E2F3 overexpression. After PPIX treatment, miR-199a-5p was modulated to increase and led to the inhibition of E2F3 expression [53]. In murine models of colon cancer, miR-143 and miR-145 played tumor suppressive functions by targeting Cdk6, CCND2 and E2F3 [54].

The miR-210 gene, located at the tip of the short arm of chromosome 11, is found to be differentially expressed in different neoplasms and consistently stimulated under hypoxic conditions [55]. It has been reported that miR-210 expression in pancreatic cancer (PC) cells was induced by hypoxia through a HIF-1α-dependent pathway, and E2F3 was identified as its potential target [56]. Gene copy aberrations of the miR-210 locus were very frequent in ovarian cancer, and its reduction could induce deregulation of the hypoxia response by targeting E2F3, which in turn promoted tumor development [57]. Interestingly, miR-210 was reported to exert its anti-apoptotic effects of human pulmonary artery smooth muscle cell (HPASMC) by targeting E2F3 [58]. In a recent research, tissue inhibitor of metalloproteinases-1 (TIMP-1) was reported to lead to a pro-tumourigenic increase of miR-210 in lung adenocarcinoma cells along with their exosomes and E2F3, a downstream target of miR-210, was decreased in the presence of TIMP-1 [59]. Similarly, in lung adenocarcinoma, miR-432 functioned as a tumor suppressor gene through targeting E2F3 and AXL [60].

The three members of miR-125 family (miR-125a, miR-125b-1 and miR-125b-2) have been found to function as either promoter or repressor in different cancers [61]. A recent study has shown that downregulation of miR-125a-5p was involved in gastric carcinogenesis by targeting E2F3 [62]. It was also reported that miR-125a-5p could function as a negative regulator of C2C12 myoblast proliferation *via* regulation of E2F3 [61]. Circulating miR-125b expression is reported to correlate with chemoresistace of breast cancer, and importantly, reduction of miR-125b could sensitize breast cancer cell to chemotherapy by targeting E2F3 [63]. In prostate cancer (PCa), miR-125b was fond to be regulated by 1,25-dihydroxyvitamin D and function as a tumor suppressor via regulation of E2F3 [64]. It has been demonstrated that miR-29a, miR-29b and miR-874 were downregulated in most of the osteosarcoma tissues and higher expression of miR-29a increased the expression of E2F1 and E2F3 [65, 66]. E2F3 was also identified as a target of miR-874 in osteosarcoma cells and E2F3 overexpression could partially reverse its tumor-suppressive effects [66].

The miR-200 family, composed of four members (miR-200a, miR-200b, miR-200c, miR-141 and miR- 429), has been reported to play roles in various celluar processes, such as growth, migration, invasion and chemoresistance [9]. It was demonstrated that miR-200c was significantly diminished in bladder cancer tissues and appeared to control the EMT process of bladder cancer cells *via* regulation of BMI-1 and E2F3 [67]. miR-141 was observed to be significantly decreased in both HCC tissues and cell lines and E2F3 was identified as its direct target [68]. Meanwhile, miR-429 was reported to exert its tumor suppressive effect in renal cell carcinoma by directly targeting BMI1 and E2F3 [69].

Actually, the exact roles of these miRNAs and E2F3 gene in tumorigenesis have not been clearly elucidated in different cancers. By combining miRNA profiling and bioinformatics tools, researchers sought not only to predict potential mechanisms but also to provide insights into the pathogenesis of cancers. It was shown that stage I epithelial ovarian cancer (EOC) histotypes had their own characteristic miRNA profiles and specific regulatory circuits. miR-30a was found to be negatively correlated with E2F3 expression, and clear cell subtype had significantly lower levels of E2F3 compared with the other subtypes [70]. In cardiovascular diseases and cancers, E2F3 was also testified as a representative target of miR-125b and miR-195 [71]. Also, transfection of miR-34c could be found to decrease E2F3 protein levels in head and neck cancer cells [72]. In ovarian cancer, one potentially biological relevance pair (hsa-miR-145/E2F3) was identified [73]. In K562 and HeLa cells, silencing of E2F3, c-Myc or Pim-1 negatively affected expression of miR-17-92 cluster [74]. Methylation of miR-152 and miR-10b-5p promoted multiple myeloma (MM) progression by upregulating expression of specific oncogenes (DNMT1, BTRC, MYCBP and E2F3) [75].

## THE LOOPS: E2F3 REGULATES MIRNAS

**Figure 2:**
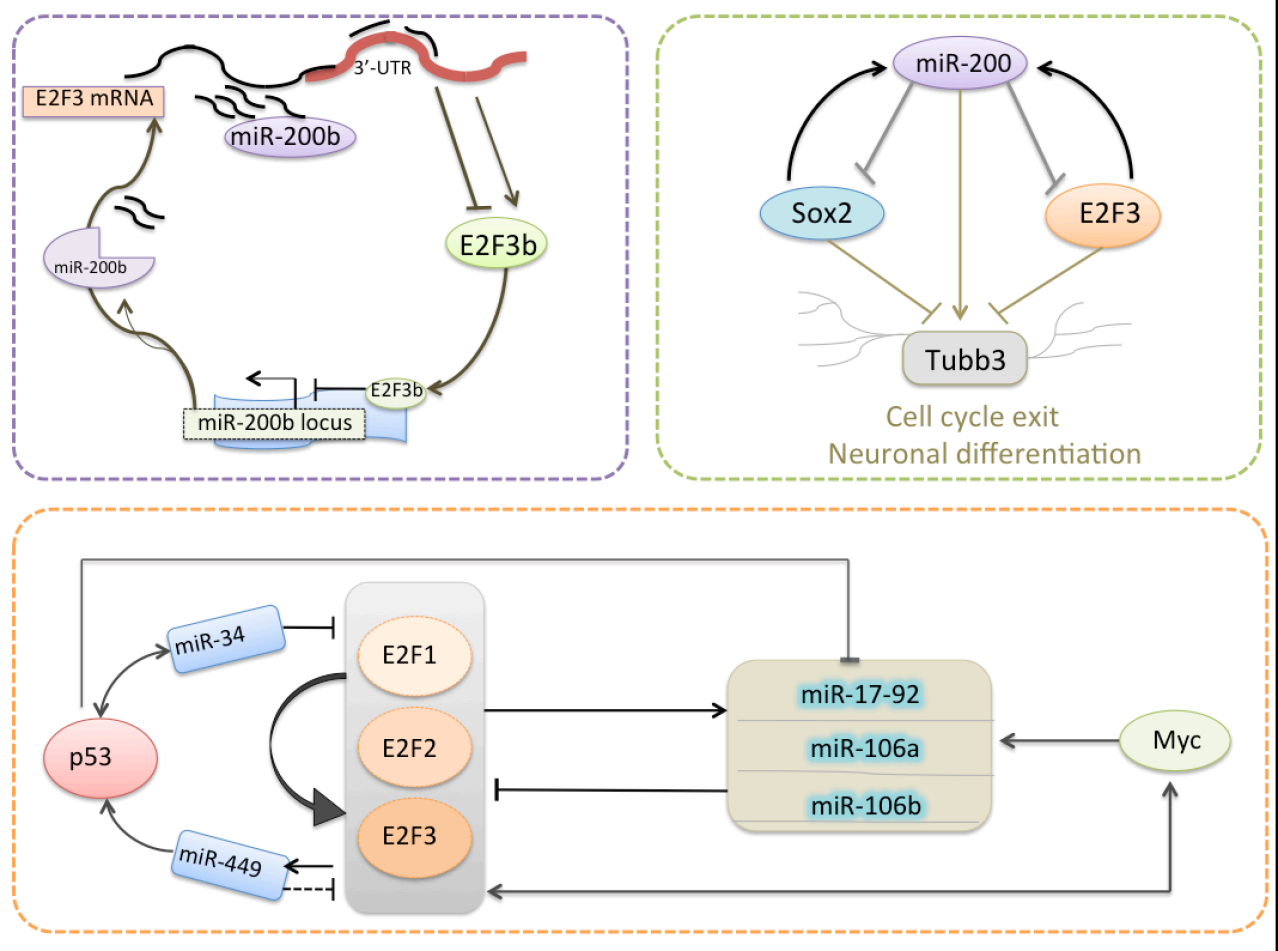
Model for several miRNAs are regulated by E2F3 in an auto-regulatory feedback loop

Reinforcing the inhibition of transcripts in specific target cells was one prominent biological function of miRNAs. Since miRNAs can be regulated by transcription factors, the regulatory loops can be established between genes coding for miRNAs and/or genes coding for classic transcription factors. As mentioned above, some miRNAs are apt to inhibit E2F3 and several of them are also regulated by E2F3 in an auto-regulatory feedback loop (Table 2; Figure 2).

**Table 2:** The loops—E2F3 regulates miRNAs

E2F3 alterations	miRNAs alterations	Cancer or cell types	Mechanism of action and validation	Cell functions	Target genes	References
E2F3	miR-200b	LAD cells	Targets 3′-UTR of E2F3 (luciferase assay)	Regulates docetaxel chemosensitivity of human LAD cells	--	[77,78]
E2F3	miR-200c	Neural stem/progenitor cells	Targets 3′-UTR of E2F3 (luciferase assay)	Governs neural progenitor cell-cycle exit and differentiation	Tubb3	[79]
E2F3	miR-17-92	Neurblastoma	Microarray experiments	--	--	[81]

Previously, we showed that miR-200b, which was identified as the most down-regulated miRNA in docetaxel-resistant SPC-A1/DTX cells, could promote docetaxel resistance in lung adenocarcinoma cells by directly targeting E2F3 [76, 77]. Moreover, E2F3 was bioinformatically identified as a potential transcriptional regulator of pre-miR-200b gene promoter, while the expression of miR-200b was confirmed to be negatively regulated by E2F3b, suggesting that there was a double-negative feedback loop between E2F3b and miR-200b [78]. The miR-200 family (mostly miR-200c) plays a critical role for the proper generation and survival of ventral neuronal populations by directly targeting the pluripotency factor Sox2 and the cell-cycle regulator E2F3 in neural stem/progenitor cells. Peng et al. showed that there was a unilateral negative feedback loop between miR-200 family and Sox2/E2F3 during neural progenitor cell-cycle exit and differentiation [79]. In particular, activation of the miR-17-92 cluster could balance the positive auto-regulatory circuit of E2F1-3 by a negative feedback loop so as to create a fail-safe mechanism against a high cell context-dependent activity of E2F1. Meanwhile, miR-449a/b and miR-34 was reported to induce inhibition of E2F1 and E2F3 in a negative feedback loopy, respectively [80]. It was reported that miR-17-5p and miR-106a was found to be up-regulated by N-Myc in human neuroblastomas, while the activation of N-Myc was revealed to be directly regulated by the E2F1-3 transcriptional regulators in neuroblastomas [81, 82]. Considering that a single miRNA is capable of affecting multiple target genes in posttranscriptional level, it is not surprising that miRNAs can be directly or indirectly restrained by their target genes, leading to the formation of complex regulatory feedback networks.

## THE POTENTIAL MOLECULAR MECHANISMS

Considering the cancer type-depending dysregulation of miRNAs/E2F3 loops, this deregulation may play an intricate role in carcinogenesis. In the following section, we will summarize the roles of these deregulated miRNAs/E2F3 loops according to their various biological functions including growth, apoptosis, cell cycle, metastasis and multiple clinical treatment steps of the tumorigenicity cascade (Figure 1–2).

### Proliferation

It is well known that the most characteristic property of tumor cells is uncontrolled cell growth. In glioma cells, overexpression of miR-128 inhibited cell proliferation in T98G cells by targeting E2F3a and augment of E2F3a can partly rescue the proliferation inhibition caused by miR-128 [19]. Likewise, down-regulation of miR-128 was reported to promote proliferation of undifferentiated GBM cells, in part, by coordinately up-regulating E2F3a [20]. In another study, a mechanism of methylation that controlled miR-128-1 expression in GBM cells and GSCs was presently revealed and miR-128-1 functioned as a tumor suppressor in GBM by directly targeting BMI1 and E2F3 [21]. In melanoma cell lines, ectopic expression of miR-377 was observed to inhibit proliferation and reduce colony formation by decreasing both E2F3 and MAP3K7 protein levels [22]. According to a recent report, it was found that long non-coding RNA-NEAT1 promoted lung cancer cell growth *in vivo* by inhibiting miR-377-3p/E2F3 axis [23]. Ectopical expression of miR-34a exerted an anti-proliferative effect by targeting E2F3 during retinoicacid-induced differentiation of the SK-N-BE cell line [26]. Importantly, an aberrant functional network comprising miR-34a/Sirt1/E2F3 was testified to promote the proliferation of the 5-FU-resistant colon cancer cells [28]. T-VISA-miR-34a induced higher miR-34a level and significantly inhibited breast cancer cell growth *in vitro* by downregulating E2F3 [30]. In HNSCC cell lines, re-expression of miR-34a could significantly reduce the capacities of cell proliferation and colony formation by downregulation of E2F3 and survivin [31]. Geng et al. showed that overexpression of miR-34a could impede HPV-positive cancer cell viability by targeting E2F3 and survivin [35]. Additionally, upregulation of either miR-497 or miR-200c inhibits proliferation of bladder cancer cells by downregulating E2F3 [40, 67]. miR-449b inhibited the cell growth and viability of SW1116 colon cancer stem cells via reduction of E2F3 [44]. The introduction of miR-449a was found to be result in cell cycle arrest and cell senescence in A549 and 95D cells by targeting E2F3 [45]. Practical reports showed that miR-503 suppressed proliferation of HCC cells through Rb-E2F signaling pathways [48]. Also, overexpression of miR-503 also remarkably inhibited CRC cell proliferation by targeting E2F3 [49]. The expression levels of miR-144, miR-141 and miR-214 were negatively correlated with E2F3 level, and overexpression of those miRNAs was found to inhibit proliferation of HCC cells [50, 68, 83]. Xu et al. showed that ectopic expression of miR-125a-5p significantly inhibited growth of gastric cancer cells by directly targeting E2F3 [62]. In addition, miR-125a-5p and E2F3 displayed reversely associated expression pattern in the proliferative phase of C2C12 myoblasts [61]. In gastric cancer cells, miR-145 mediated the anti-proliferative effects of vitamin D3 by directly targeting E2F3 [47]. In renal carcinoma, miR-429 inhibited cell proliferation by negatively regulating both BMI1 and E2F3 [69]. In lung adenocarcinoma, overexpression of miR-432 inhibited cell proliferation through arresting cell cycle by regulation of two directly targets E2F3 and AXL [60]. Overexpression of miR-874 was reported to remarkably inhibit proliferation and metastasis of osteosarcoma by targeting E2F3 [66]. Previously, we also showed that re-expression of miR-200b could reverse docetaxel chemoresistance of lung adenocarcinoma cells through growth inhibition by targeting E2F3 [76]. Our further research indicated that there was a double-negative feedback loop between E2F3b and miR-200b which was involved in regulation of DTX chemosensitivity of lung adenocarcinoma cells [78]. It was found that the miR-200 family (in particular miR-200c) stimulated cell-cycle distribution and neuronal differentiation of vMH neural progenitors by down-regulating expression of Sox2 and E2F3 [79].

### Cell cycle

It is generally considered that E2F3 transcription factor drives cell cycle G1 to S phase. E2F3a was important for DNA synthesis and cell cycle progression, while E2F3b was expressed throughout the cell cycle [85]. It has been reported that miR-34a acted as a tumor suppressor in AML by inhibiting E2F3, resulting in blocking the transition from cell cycle G1 to S phase [24]. In another report, it was found that the expression of miR-34a which was putatively involved in G1 arrest and apoptosis could be directly induced by p53 [86]. Interestingly, inactivating p53 mutations were associated with higher levels of E2F3a in ovarian cancer, and lower miR-34a levels in p53 mutated tumors could be observed than those in p53 wild-type tumors [25]. Also, E2F3 was confirmed to be down-regulated upon induction of miR-210 in ovarian cancer and miR-210 linked hypoxia with regulation of cell cycle and played a crucial role in ovarian cancer onset [57]. According to a recent report, it was shown that overexpression of miR-34c significantly inhibited proliferation of endometrial carcinoma cells by reducing E2F3 [36]. In malignant melanoma, miR-203 was reported to induce senescence by cell cycle arrest through targeting E2F3 [41]. EBV down-regulated miR-203 through the oncoprotein LMP1, which contributed to higher incidence of tumor in epithelial cells. Further data indicated that re-expression of miR-203 could inhibit EBV-induced S-phase entry and *in vivo* transformation *via* regulating E2F3 [43]. In colon cancer, transfection of miR-449b precursor could significantly increase the number of cells in the G0-G1 phase *via* regulation of cell cycle function of E2F3 [44]. Meanwhile, enforced miR-449a could lead to cell cycle arrest and cell senescence *via* regulation of E2F3 expression in lung cancer cells [45]. In gastric cancer cells, significant G0/G1 arrest was also observed after transfection of miR-449a mimics, which were stimulated by directly targeting of E2F3 [46]. Overexpression of miR-145 was also found to inhibit gastric cancer cell proliferation through regulation of E2F3-dependent cell cycle transition [47]. Another study showed that miR-503 induced G1 phase arrest of cell cycle *via* downregulation of cyclin D3 and E2F3 in HCC cells [48]. Meanwhile, miR-503 performed similar performance in CRC cells by directly targeting E2F3 [49]. Likewise, miR-214 was found to induce G1-S phase arrest by suppressing E2F3-dependent cell cycle regulation [83]. Other findings indicated that EGFR signals could coordinately control multiple G1 regulators *via* miR-143 and its target K-ras and miR-145 and its targets MYC, cdk6, CCND2 or E2F3, which play a role in cell cycle progression in colon cancer [54]. Augment of miR-125a-5p affected DNA replication and promoted G1 arrest by regulating E2F3 [61]. E2F3 was also identified as a functional downstream target of miR-195 *via* robust arrested cell cycle progression in glioblastoma cells [39]. miR-29 could affect expression of E2F3 and lead to the cell cycle arrest in osteosarcoma [65]. Our previous study have shown that enforcement of miR-200b reversed docetaxel chemoresistance of lung adenocarcinoma cells by inducing G2/M cell cycle arrest through targeting E2F3 [76]. In further research, we confirmed that double-negative feedback loop between E2F3b and miR-200b could regulate the sensitivity of lung adenocarcinoma cells to docetaxel through cell cycle distribution [78].

### Apoptosis

It is well known that apoptosis is a process of programmed cell death which occurs in multi-cellular organisms. Excessive apoptosis causes atrophy, whereas an insufficient amount results in uncontrolled cell proliferation, such as cancer. Augmented miR-377-3p significantly promoted cell apoptosis inhibited by over-expressed-NEAT1 treatment. E2F3 over-expression remarkably reversed the effect of miR-377-3p on reduction of the caspase-3/7, also reversed the suppressive role of miR-377-3p on anti-apoptotic protein Bcl-2 and promotion of cleaved-caspase-3 protein expression. These results confirmed that miR-377-3p/E2F3 made sense in NEAT1-induced inhibitory roles on NSCLC cells [23]. miR-34a was demonstrated to be a tumor suppressor with anti-proliferative effects in a caspase 3/7 apoptotic pathway in NB cell lines [26]. Li F et al. suggested that miR-34c acted as a tumor suppressor *via* induction of apoptosis *via* E2F3 [36]. Also, re-expression of miR-449a significantly increased apoptosis of gastric cancer cells by targeting E2F3 [46]. Likewise, miR-503 was found to induce apoptosis by targeting E2F3 [49]. miR-210, a hypoxia-inducible miRNA, inhibited apoptosis of HPASMC cells in hypoxia by downregulating E2F3 [58]. Zhang et al. showed that miR-29a regulate apoptosis of osteoblastic cell by downregulation of Bcl-2 and Mcl-1 and upregulation of E2F1 and E2F3 [65]. And, miR-874 inhibited osteosarcoma cell growth by inducing cell apoptosis through downregulation of E2F3 [66]. Woods et al. showed that miR-17-92 contributed to cell proliferation by shifting the E2F transcriptional balance away from the pro-apoptotic E2F1 and toward the proliferative E2F3 transcriptional network [87]. It was also confirmed that miR-125 and miR-106a were upstream regulators of E2F3 and RB1, respectively, *via* the mechanisms involved cisplatin-induced K562 cell apoptosis [88]. Ectopic miR-200b/E2F3 feed loop could reverse docetaxel chemoresistance of LAD cells *via* cell apoptosis enhancement [76].

### Angiogenesis

Angiogenesis, a normal and vital process in growth and development, is known to be a fundamental step in the transition of tumors from a benign state to a malignant one. Kumar B et al. listed the main features of miR-34a as follows, overexpression of miR-34a inhibited tumor angiogenesis by reducing VEGF production by head and neck squamous cell carcinoma cells as well as directly inhibiting endothelial cell functions, and E2F3a was identified its target gene [31]. More interestingly, TIMP-1 was able to increase miR-210 in tumor cell and in exosomes by induction of PI3K/AKT/HIF-1 signaling, and this increase of miR-210 in exosomes displayed a pro-angiogenic effect *in vitro*. Downstream targets of miR-210, namely FGFRL1, E2F3, VMP-1, RAD52 and SDHD, were declined in the presence of TIMP-1. It was finally identified that TIMP-1-induced PI3K/AKT/HIF-1/miR-210/EphA3 signaling displayed pro-angiogenic properties [59]. In HCC, PPIX was testified to inhibit mesenchymal tumor angiogenesis, depending on the increase of miR-199a-5p by targeting E2F3 [53].

### Invasion and metastasis

It is well known that metastasis is the spread of tumor from one organ or part of the body to another without being directly connected with it and with uncontrolled growth, which allows for invasion into the circulation, followed by invasion to a second site for tumorigenesis [89]. Either forced expression of miR-128-1 or Aza/PBA treatment inhibited migration and invasion of tumor cells *in vitro*, and E2F3 was found to be a direct target of and downregulated by miR128-1 *in vitro* and *in vivo* [21]. Initial observations suggested a link between miR-377 and E2F3, and ectopic miR-377 expression decreased migratory capacity in melanoma cells by targeting E2F3 [22]. Up-regulated miR-377-3p in NSCLC cells, which stably over-expressed NEAT1, significantly reversed the favorable roles of NEAT1 on metastasis. miR-377-3p exerted tumor suppressive function through down-regulation of E2F3 [23]. T-VISA-miR-34a, which increased miR-34a expression, significantly suppressed breast cancer migration and invasion *in vitro* by downregulating E2F3 [30]. Meanwhile, it was also found that overexpression of miR-34a significantly inhibited tumor migration in HNSCC cell lines *via* downregulation of E2F3 and surviving [31]. Upregulation of miR-34c significantly inhibited migration and invasion of HEC1-B cells *via* reduction of E2F3 protein [36]. Silencing of miR-497 enhanced the migration and invasion of BTCC cells through upregulation of E2F3 and subsequently facilitated the development of BTCC, and the rescue effect of E2F3 expression partly reversed the inhibition of migration and invasion induced by miR-497 [40]. Several other studies revealed the potential roles of miR-200c in bladder cancer metastasis, and overexpression of miR-200c significantly inhibited invasion and migration of bladder cancer cells and led to conspicuous reduction of BMI-1 and E2F3 [67]. miR-203 could inhibit glioma cells invasion by target E2F3 [42]. Studies demonstrated the biological functions of miR-144, miR-141 and miR-217, with the ability to suppress migration and invasion of HCC cells by downregulating E2F3 [50, 51, 68]. Ectopic up-regulation of miR-199a-5p/E2F3 contributed to the inhibition of invasion and migration in HCC, accounting for the anti-tumor effect of PPIX [53]. In gastric cancer, suppression of miR-125a-5p was observed to promote tumor metastasis, while upregulation of miR-125a-5p substantially reduced the capacities of migration and invasion [62]. Also, re-expression of miR-874 could remarkably suppress migration and invasion of osteosarcoma cells by targeting E2F3. And, the role of miR-429 in metastatic process of RCC cells was explored, and a direct association between miR-429 and E2F3 was identified [69].

## CLINICAL APPLICATION

### Cancer diagnosis and prognosis

Ongoing efforts would be made in identifying new biomarkers to help tumor early diagnosis. Moreover, the poorer prognosis of tumor patients is mainly caused by higher tendency of tumor invasiveness, resistance to anticancer therapies and higher frequency of tumor recurrence. It was suggested that reduced miR-497 was associated with poorer prognosis of BTCC patients and miR-497/E2F3 axis may be valuable biomarkers for BTCC progression [40]. Also, downregulation of miR-449a was observed to be highly associated with tumor recurrence and lung cancer patients' survival, suggesting that miR-449a/E2F3 played a critical role in development of lung cancer [45]. Reduced miR-503 was observed to be correlated with worse overall survival of HCC patients and enhanced malignant potential, including portal vein tumor thrombi, histologic grade, TNM stage and AFP level [48]. Moreover, reduction of miR-424 was found to be associated with poor prognosis of HCC patients, suggesting that miR-424 could be a valuable biomarker for HCC prognosis [52]. It was also reported that downregulation of miR-432 was correlated with a higher clinical stage and poorer prognosis in lung adenocarcinoma patients [60].

Recently, it was demonstrated that EGFR/miR-145/E2F3 pathway could offer a promising strategy for tumor prevention in high-risk individuals [54]. During the early stages of EBV latency, miR-203 was observed to be decreased in EBV-infected epithelial cells and EBV-associated NPC, occurring, implying that EBV-LMP1 downregulated miR-203 and ulteriorly controlled E2F3. Furthermore, EBV-LMP1/miR-203/E2F3 axis may be used as a potential biomarker for the diagnosis and therapeutic agent of EBV-associated NPC [43]. One study showed that miR-29 played a critical role in osteosarcoma pathogenesis, while other studies provided evidence that miR-874 expression was correlated with TNM stage, tumor size, and lymph node metastasis. Thus, these data indicated that miR-29 and miR-874 mediated network involving in E2F3 may be employed as a prognosis marker a novel candidate for osteosarcoma therapeutics [65, 66]. In renal cell carcinoma, miR-429 was found to function as a tumor suppressor *via* regulation of E2F3, suggesting this potential development of miR-429-based prognostic and therapeutic approaches may be valuable for the treatment of renal cell carcinoma patients [69].

### Cancer treatment

Resistance of tumor cells to chemotherapy often leads to the subsequent tumor recurrence and metastasis. Therefore, it is needed to investigate the molecular mechanisms involved in tumor chemoresistance so as to develop molecular markers to predict clinic treatment outcome and explore novel strategies for reversing this chemoresistance. Akao et al. showed that re-expression of miR-34a obviously reversed the 5-FU chemoresistance of colorectal cancer cells *via* down-regulation of Sirt1 and E2F3 [28]. A current result reported that oxaliplatin led to early p53 accumulation, increased expression of miR-34a, and subsequent reduction of E2F3 and E2F1 and 1,2-dia-minocyclohexane carrier ligand in oxaliplatin triggers the p53-miR-34a-E2F3-E2F1 axis, resulting in transcriptional regulation that finally induced accumulation of dUTP and decreased dTTP biosynthesis, potentially increasing 5-FU sensitivity [29]. Another report showed that miR-203 was positively correlated with the sensitivity of glioma cells to chemotherapy and re-expression of miR-203 increased glioma cells to temozolomide by targeting E2F3 [42]. A foregoing data indicated that the synergistic role of PPIX increased in the anti-cancer efficacy of doxorubicin or cisplatin in mesenchymal liver tumor cells. PPIX, regulating miR-199a-5p/E2F3, prevented tumor cell growth and migration of tumor cells and sensitized mesenchymal hepatoma cells to chemotherapeutic agents [53]. Also, it was observed that reduction of miR-125b increased the chemosensitivity of breast cancer cells to 5-FU by targeting E2F3 and high miR-125b expression was correlated with poor clinical response of breast cancer patients to chemotherapy, suggesting that circulating miR-125b levels had potential of being used as a therapeutic target for reversing clinical chemoresistance [63]. Furthermore, depressed miR-432 could promote the disease progression, especially the drug resistance, though E2F3 and AXL. The findings highlighted the meaning of miR-432 dysfunction in promoting chemoresistance and implicated miR-432 as a promising molecular target for treating lung cancer [60]. A novel study suggested that 1,25(OH)2D3 inhibited proliferation of gastric cancer cells *via* regulation of miR-145 and its target E2F3 and targeting vitamin D/miR-145/E2F3 pathway will be a potential strategy for gastric cancer treatment [47].

It has been accepted that miRNAs act as master regulators of key cellular pathways in human cancers. The findings suggested that miR-128-1 could impede the growth of glioblastoma and GSCs *via* targeting BMI1 and E2F3, so miR-128-1 and DNA demethylating agents were promising for anti-glioma therapy at least in part by eliminating GSCs [21]. Another work showed that miR-377 was a key regulator of E2F3 and MAP3K7/NF-κB pathways in melanoma progression, rendering it a potential new therapeutic target in melanoma [22]. Another finding showed that miR-34a was frequently dysregulated in HNSCC and targeting miR-34a-E2F3a-survivin axis might be a promising approach for HNSCC therapy [31]. The glad news was put forward that miR-203 may function as a tumor suppressor in glioma progression, suggesting that targeting miR-203/E2F3 axis could be a rational therapeutic strategy for glioma [42]. Additionally, dysregulation of miR-449b/E2F3 signal plays a role in the self-renewal and proliferation of colon cancer stem cell and targeting this signal will be a potential strategy for the treatment of colon cancer [44]. Moreover, miR-503 functioned as a tumor suppressor in HCC by directly targeting cyclin D3 and E2F3 through Rb-E2F signaling pathway, suggesting that miR-503/E2F3 may act as helpful therapeutic targets for HCC therapy [48]. Meanwhile, miR-217 was reported to function as a tumor suppressor in HCC progression and miR-217/E2F3 and miR-214/E2F3 axises may be potential candidates for developing rational therapeutic approaches [51, 83]. Since data demonstrated the roles of miR-424/Akt3/E2F3 axis in HCC development, this identification would be helpful to better elucidation of the molecular mechanisms and carry implications in HCC intervetion/prevention and treatment [52].

Our previous results showed for the first time that miR-200b could be used as a restorer of chemosensitivity of lung adenocarcinoma cells to docetaxel, mediated at least partially by targeting E2F3 [76]. Afterward, we proved a novel mechanism in which E2F3b and miR-200b each suppressed the other, suggesting a novel functional double-negative feedback loop. Artificial controls on this circuit may be potential reversal strategies in overcoming chemoresistance of lung cancer [78].

## CONCLUSIONS AND PERSPECTIVES

Here, we briefly summarized the bidirectional interactions between E2F3 and miRNAs in human cancers. Many miRNAs can restrain the activity of E2F3 family members, and E2F3 can in turn regulate many different miRNAs, which play roles in various biological processes that are intertwined with the control of tumor pathogenesis and progression. Recent findings have outlined the tight connection between E2F3 activity and miRNA expressions. Present studies have provided additional evidences with respect to miRNAs post-transcriptionally regulating target genes *via* imperfect pairing with the 3′-UTR of their target mRNAs, leding to degradation or translational repression of mRNAs in most cases. However, the causes of dysregulated miRNAs in human cancers are not fully elucidated, and some reasons (genetic abnormalities, epigenetic regulation, post-transcriptional modulation, etc) have been pronounced [90].

E2F3, a member of the transcription factor E2F family, has two distinct isoforms: E2F3a and E2F3b [4]. E2F3a belongs to transcriptional activators, which is critical in the control of cell proliferation by releasing from pRb, and E2F3b is repressors that could restrain the transcription of E2F target genes when cooperate with the pocket proteins p107 and p130 and with the addition of repressive histone deacetylases during the early G1 phase. E2F3, E2F3a and E2F3b could act polytropic roles, which exert cooperative effects or opposed effects in tumorigenesis and tumor progression. Groups of tumor cells possess the intrinsic and highly ordered development ability of resistance, whether cytotoxic drugs or targeting drugs have failed to overcome the problem of drug resistance. Drug resistance is the main reasons affecting the curative effect and poor prognosis. Apoptosis pathway abnormality not only is a reason of cell malignant transformation, but also is the principal mechanisms of drug resistance. It was well known that Rb and p53 are two significant promoting apoptosis proteins. And the sensitivity of tumor cells to chemotherapy drugs disparities among in different phase of cell cycle. As mentioned, E2F3 regulates chemosensitivity of human cancers mainly through cell proliferation, cell cycle distribution and apoptosis. These signal pathways and feedback loops would be the potential mechanism for reversing chemoresistance. Thus, it is necessary to accept that the importance of miRNAs/E2F3 regulation network in various signal pathways, which might contribute to developing more specific therapeutic targets and drugs. To date, the transcription factors which positively or negatively regulate expression of miRNAs in a tissue-specific or development-specific manner have been reported one by one. We believe that, except for miR-200, miR-17 and miR-106, other miRNAs may also form negative feedback loops with E2F3, which needs to be further explored. Likewise, other transcription factors (CREB1, bHLH-PAS and Twist1) are also clarified to exist the reciprocal links with miRNAs [91–93]. Further study on the cross-link between E2Fs and miRNAs may be helpful to further understand the functions of E2Fs in specific conditions. Taken together, E2F3 regulates the expression of not only protein-coding genes but also miRNAs. Both E2F3 and miRNAs have been found to control important cancer hallmarks, such as growth, cell cycle, apoptosis, angiogenesis, metastasis, and chemoresistance. E2F3/miRNA axis positively regulates cellular biological activity of tumor, pointing to a regulatory network of high complexity in the functional net effect of the multiple miRNAs. The crosstalk between miRNAs and E2F3 would provide new potential diagnostic and therapeutic strategies in cancer treatment.

## References

[1] DeGregori J (2002). The genetics of the E2F family of transcription factors: shared functions and unique roles. Biochim Biophys Acta.

[2] Stanelle J, Pützer BM (2006). E2F1-induced apoptosis: turning killers into therapeutics. Trends Mol Med.

[3] Maiti B, Li J, de Bruin A, Gordon F, Timmers C, Opavsky R, Patil K, Tuttle J, Cleghorn W, Leone G (2005). Cloning and characterization of mouse E2F8, a novel mammalian E2F family member capable of blocking cellular proliferation. J Biol Chem.

[4] Adams MR, Sears R, Nuckolls F, Leone G, Nevins JR (2000). Complex transcriptional regulatory mechanisms control expression of the E2F3 locus. Mol Cell Biol.

[5] Martinez LA, Goluszko E, Chen HZ, Leone G, Post S, Lozano G, Chen Z, Chauchereau A (2010). E2F3 is a mediator of DNA damage-induced apoptosis. Mol Cell Biol.

[6] Nahle Z, Polakoff J, Davuluri RV, McCurrach ME, Jacobson MD, Narita M, Zhang MQ, Lazebnik Y, Bar-Sagi D, Lowe SW (2002). Direct coupling of the cell cycle and cell death machinery by E2F. Nat Cell Biol.

[7] Engelmann D, Knoll S, Ewerth D, Steder M, Stoll A, Pützer BM (2010). Functional interplay between E2F1 and chemotherapeutic drugs defines immediate E2F1 target genes crucial for cancer cell death. Cell Mol Life Sci.

[8] Nicoloso MS, Spizzo R, Shimizu M, Rossi S, Calin GA (2009). MicroRNAs—the micro steering wheel of tumour metastases. Nat Rev Cancer.

[9] Gao Y, Feng B, Han S, Zhang K, Chen J, Li C, Wang R, Chen L (2016). The Roles of MicroRNA-141 in Human Cancers: From Diagnosis to Treatment. Cell Physiol Biochem.

[10] Calin GA, Sevignani C, Dumitru CD, Hyslop T, Noch E, Yendamuri S, Shimizu M, Rattan S, Bullrich F, Negrini M, Croce CM (2004). Human microRNA genes are frequently located at fragile sites and genomic regions involved in cancers. Proc Natl Acad Sci USA.

[11] Chaudhuri K, Chatterjee R (2007). MicroRNA detection and target prediction: integration of computational and experimental approaches. DNA Cell Biol.

[12] Bracken CP, Gregory PA, Kolesnikoff N, Bert AG, Wang J, Shannon MF, Goodall GJ (2008). A double-negative feedback loop between ZEB1-SIP1 and the microRNA-200 family regulates epithelial-mesenchymal transition. Cancer Res.

[13] Kefas B, Comeau L, Floyd DH, Seleverstov O, Godlewski J, Schmittgen T, Jiang J, diPierro CG, Li Y, Chiocca EA, Lee J, Fine H, Abounader R (2009). The neuronal microRNA miR-326 acts in a feedback loop with notch and has therapeutic potential against brain tumors. J Neurosci.

[14] Pulikkan JA, Dengler V, Peramangalam PS, Peer Zada AA, Müller-Tidow C, Bohlander SK, Tenen DG, Behre G (2010). Cell-cycle regulator E2F1 and microRNA-223 comprise an autoregulatory negative feedback loop in acute myeloid leukemia. Blood.

[15] Wu H, Wang G, Wang Z, An S, Ye P, Luo S (2015). Anegative feedback loop between miR-200b and the NF-kappaB pathway via IKBKB/IKK-beta in breast cancer cells. FEBS J.

[16] Zhao H, Kalota A, Jin S, Gewirtz AM (2009). The c-myb proto-oncogene and microRNA-15a comprise an active autoregulatory feedback loop in human hematopoietic cells. Blood.

[17] Jing J, Xiong S, Li Z, Wu J, Zhou L, Gui JF, Mei J (2015). A feedback regulatory loop involving p53/miR-200 and growth hormone endocrine axis controls embryo size of zebrafish. Sci Rep.

[18] Peter ME (2010). Targeting of mRNAs by multiple miRNAs: the next step. Oncogene.

[19] Zhang Y, Chao T, Li R, Liu W, Chen Y, Yan X, Gong Y, Yin B, Liu W, Qiang B, Zhao J, Yuan J, Peng X (2009). MicroRNA-128 inhibits glioma cells proliferation by targeting transcription factor E2F3a. J Mol Med (Berl).

[20] Cui JG, Zhao Y, Sethi P, Li YY, Mahta A, Culicchia F, Lukiw WJ (2010). Micro-RNA-128 (miRNA-128) down-regulation in glioblastoma targets ARP5 (ANGPTL6), Bmi-1 and E2F-3a, key regulators of brain cell proliferation. J Neurooncol.

[21] Shan ZN, Tian R, Zhang M, Gui ZH, Wu J, Ding M, Zhou XF, He J (2016). miR128-1 inhibits the growth of glioblastoma multiforme and glioma stem-like cells via targeting BMI1 and E2F3. Oncotarget.

[22] Zehavi L, Schayek H, Jacob-Hirsch J, Sidi Y, Leibowitz-Amit R, Avni D (2015). MiR-377 targets E2F3 and alters the NF-kB signaling pathway through MAP3K7 in malignant melanoma. Mol Cancer.

[23] Sun C, Li S, Zhang F, Xi Y, Wang L, Bi Y, Li D (2016). Long non-coding RNA NEAT1 promotes non-small cell lung cancer progression through regulation of miR-377-3p-E2F3 pathway. Oncotarget.

[24] Pulikkan JA, Peramangalam PS, Dengler V, Ho PA, Preudhomme C, Meshinchi S, Christopeit M, Nibourel O, Müller-Tidow C, Bohlander SK, Tenen DG, Behre G (2010). C/EBPα regulated microRNA-34a targets E2F3 during granulopoiesis and is down-regulated in AML with CEBPA mutations. Blood.

[25] Reimer D, Hubalek M, Kiefel H, Riedle S, Skvortsov S, Erdel M, Hofstetter G, Concin N, Fiegl H, Müller-Holzner E, Marth C, Altevogt P, Zeimet AG (2011). Regulation of transcription factor E2F3a and its clinical relevance in ovarian cancer. Oncogene.

[26] Welch C, Chen Y, Stallings RL (2007). MicroRNA-34a functions as a potential tumor suppressor by inducing apoptosis in neuroblastoma cells. Oncogene.

[27] Cho WC (2007). OncomiRs: the discovery and progress of microRNAs in cancers. Mol Cancer.

[28] Akao Y, Noguchi S, Iio A, Kojima K, Takagi T, Naoe T (2011). Dysregulation of microRNA-34a expression causes drug-resistance to 5-FU in human colon cancer DLD-1 cells. Cancer Lett.

[29] Kiyonari S, Iimori M, Matsuoka K, Watanabe S, Morikawa-Ichinose T, Miura D, Niimi S, Saeki H, Tokunaga E, Oki E, Morita M, Kadomatsu K, Maehara Y, Kitao H (2015). The 1,2-Diaminocyclohexane Carrier Ligand in Oxaliplatin Induces p53-Dependent Transcriptional Repression of Factors Involved in Thymidylate Biosynthesis. Mol Cancer Ther.

[30] Li L, Xie X, Luo J, Liu M, Xi S, Guo J, Kong Y, Wu M, Gao J, Xie Z, Tang J, Wang X, Wei W (2012). Targeted expression of miR-34a using the T-VISA system suppresses breast cancer cell growth and invasion. Mol Ther.

[31] Kumar B, Yadav A, Lang J, Teknos TN, Kumar P (2012). Dysregulation of microRNA-34a expression in head and neck squamous cell carcinoma promotes tumor growth and tumor angiogenesis. PLoS One.

[32] Altieri DC (2003). Survivin, versatile modulation of cell division and apoptosis in cancer. Oncogene.

[33] Fukuda S, Pelus LM (2006). Survivin, a cancer target with an emerging role in normal adult tissues. Mol Cancer Ther.

[34] Jiang Y, Saavedra HI, Holloway MP, Leone G, Altura RA (2004). Aberrant regulation of survivin by the RB/E2F family of proteins. J Biol Chem.

[35] Geng D, Song X, Ning F, Song Q, Yin H (2015). MiR-34a Inhibits Viability and Invasion of Human Papillomavirus-Positive Cervical Cancer Cells by Targeting E2F3 and Regulating Survivin. Int J Gynecol Cancer.

[36] Li F, Chen H, Huang Y, Zhang Q, Xue J, Liu Z, Zheng F (2015). miR-34c plays a role of tumor suppressor in HEC-1-B cells by targeting E2F3 protein. Oncol Rep.

[37] Tsuruta T, Kozaki K, Uesugi A, Furuta M, Hirasawa A, Imoto I, Susumu N, Aoki D, Inazawa J (2011). miR-152 is a tumor suppressor microRNA that is silenced by DNA hypermethylation in endometrial cancer. Cancer Res.

[38] Porrello ER, Johnson BA, Aurora AB, Simpson E, Nam YJ, Matkovich SJ, Dorn GW, van Rooij E, Olson EN (2011). MiR-15 family regulates postnatal mitotic arrest of cardiomyocytes. Circ Res.

[39] Zhang QQ, Xu H, Huang MB, Ma LM, Huang QJ, Yao Q, Zhou H, Qu LH (2012). MicroRNA-195 plays a tumor-suppressor role in human glioblastoma cells by targeting signaling pathways involved in cellular proliferation and invasion. Neuro-oncol.

[40] Zhang Y, Zhang Z, Li Z, Gong D, Zhan B, Man X, Kong C (2016). MicroRNA-497 inhibits the proliferation, migration and invasion of human bladder transitional cell carcinoma cells by targeting E2F3. Oncol Rep.

[41] Noguchi S, Mori T, Otsuka Y, Yamada N, Yasui Y, Iwasaki J, Kumazaki M, Maruo K, Akao Y (2012). Anti-oncogenic microRNA-203 induces senescence by targeting E2F3 protein in human melanoma cells. J Biol Chem.

[42] Tang G, Wu J, Xiao G, Huo L (2015). MiR-203 sensitizes glioma cells to temozolomide and inhibits glioma cell invasion by targeting E2F3. Mol Med Rep.

[43] Yu H, Lu J, Zuo L, Yan Q, Yu Z, Li X, Huang J, Zhao L, Tang H, Luo Z, Liao Q, Zeng Z, Zhang J, Li G (2012). Epstein-Barr virus downregulates microRNA 203 through the oncoprotein latent membrane protein 1: a contribution to increased tumor incidence in epithelial cells. J Virol.

[44] Fang Y, Gu X, Li Z, Xiang J, Chen Z (2013). miR-449b inhibits the proliferation of SW1116 colon cancer stem cells through downregulation of CCND1 and E2F3 expression. Oncol Rep.

[45] Ren XS, Yin MH, Zhang X, Wang Z, Feng SP, Wang GX, Luo YJ, Liang PZ, Yang XQ, He JX, Zhang BL (2014). Tumor-suppressive microRNA-449a induces growth arrest and senescence by targeting E2F3 in human lung cancer cells. Cancer Lett.

[46] Li X, Li H, Zhang R, Liu J, Liu J (2015). MicroRNA-449a inhibits proliferation and induces apoptosis by directly repressing E2F3 in gastric cancer. Cell Physiol Biochem.

[47] Chang S, Gao L, Yang Y, Tong D, Guo B, Liu L, Li Z, Song T, Huang C (2015). miR-145 mediates the antiproliferative and gene regulatory effects of vitamin D3 by directly targeting E2F3 in gastric cancer cells. Oncotarget.

[48] Xiao F, Zhang W, Chen L, Chen F, Xie H, Xing C, Yu X, Ding S, Chen K, Guo H, Cheng J, Zheng S, Zhou L (2013). MicroRNA-503 inhibits the G1/S transition by downregulating cyclin D3 and E2F3 in hepatocellular carcinoma. J Transl Med.

[49] Chang SW, Yue J, Wang BC, Zhang XL (2015). miR-503 inhibits cell proliferation and induces apoptosis in colorectal cancer cells by targeting E2F3. Int J Clin Exp Pathol.

[50] Cao T, Li H, Hu Y, Ma D, Cai X (2014). miR-144 suppresses the proliferation and metastasis of hepatocellular carcinoma by targeting E2F3. Tumour Biol.

[51] Su J, Wang Q, Liu Y, Zhong M (2014). miR-217 inhibits invasion of hepatocellular carcinoma cells through direct suppression of E2F3. Mol Cell Biochem.

[52] Yang H, Zheng W, Shuai X, Chang RM, Yu L, Fang F, Yang LY (2015). MicroRNA-424 inhibits Akt3/E2F3 axis and tumor growth in hepatocellular carcinoma. Oncotarget.

[53] Lee JM, Heo MJ, Lee CG, Yang YM, Kim SG (2015). Increase of miR-199a-5p by protoporphyrin IX, a photocatalyzer, directly inhibits E2F3, sensitizing mesenchymal tumor cells to anti-cancer agents. Oncotarget.

[54] Zhu H, Dougherty U, Robinson V, Mustafi R, Pekow J, Kupfer S, Li YC, Hart J, Goss K, Fichera A, Joseph L, Bissonnette M (2011). EGFR signals downregulate tumor suppressors miR-143 and miR-145 in Western diet-promoted murine colon cancer: role of G1 regulators. Mol Cancer Res.

[55] Fasanaro P, D'Alessandra Y, Di Stefano V, Melchionna R, Romani S, Pompilio G, Capogrossi MC, Martelli F (2008). MicroRNA-210 modulates endothelial cell response to hypoxia and inhibits the receptor tyrosine kinase ligand Ephrin-A3. J Biol Chem.

[56] Chen WY, Liu WJ, Zhao YP, Zhou L, Zhang TP, Chen G, Shu H (2012). Induction, modulation and potential targets of miR-210 in pancreatic cancer cells. Hepatobiliary Pancreat Dis Int.

[57] Giannakakis A, Sandaltzopoulos R, Greshock J, Liang S, Huang J, Hasegawa K, Li C, O'Brien-Jenkins A, Katsaros D, Weber BL, Simon C, Coukos G, Zhang L (2008). miR-210 links hypoxia with cell cycle regulation and is deleted in human epithelial ovarian cancer. Cancer Biol Ther.

[58] Gou D, Ramchandran R, Peng X, Yao L, Kang K, Sarkar J, Wang Z, Zhou G, Raj JU (2012). miR-210 has an antiapoptotic effect in pulmonary artery smooth muscle cells during hypoxia. Am J Physiol Lung Cell Mol Physiol.

[59] Cui H, Seubert B, Stahl E, Dietz H, Reuning U, Moreno-Leon L, Ilie M, Hofman P, Nagase H, Mari B, Krüger A (2015). Tissue inhibitor of metalloproteinases-1 induces a pro-tumourigenic increase of miR-210 in lung adenocarcinoma cells and their exosomes. Oncogene.

[60] Chen L, Kong G, Zhang C, Dong H, Yang C, Song G, Guo C, Wang L, Yu H (2016). MicroRNA-432 functions as a tumor suppressor gene through targeting E2F3 and AXL in lung adenocarcinoma. Oncotarget.

[61] Sun YM, Lin KY, Chen YQ (2013). Diverse functions of miR-125 family in different cell contexts. J Hematol Oncol.

[62] Xu Y, Huang Z, Liu Y (2014). Reduced miR-125a-5p expression is associated with gastric carcinogenesis through the targeting of E2F3. Mol Med Rep.

[63] Wang H, Tan G, Dong L, Cheng L, Li K, Wang Z, Luo H (2012). Circulating MiR-125b as a marker predicting chemoresistance in breast cancer. PLoS One.

[64] Giangreco AA, Vaishnav A, Wagner D, Finelli A, Fleshner N, Van der Kwast T, Vieth R, Nonn L (2013). Tumor suppressor microRNAs, miR-100 and -125b, are regulated by 1,25-dihydroxyvitamin D in primary prostate cells and in patient tissue. Cancer Prev Res (Phila).

[65] Zhang W, Qian JX, Yi HL, Yang ZD, Wang CF, Chen JY, Wei XZ, Fu Q, Ma H (2012). The microRNA-29 plays a central role in osteosarcoma pathogenesis and progression. Mol Biol (Mosk).

[66] Dong D, Gong Y, Zhang D, Bao H, Gu G (2016). miR-874 suppresses the proliferation and metastasis of osteosarcoma by targeting E2F3. Tumour Biol.

[67] Liu L, Qiu M, Tan G, Liang Z, Qin Y, Chen L, Chen H, Liu J (2014). miR-200c inhibits invasion, migration and proliferation of bladder cancer cells through down-regulation of BMI-1 and E2F3. J Transl Med.

[68] Xue J, Niu YF, Huang J, Peng G, Wang LX, Yang YH, Li YQ (2014). miR-141 suppresses the growth and metastasis of HCC cells by targeting E2F3. Tumour Biol.

[69] Qiu M, Liang Z, Chen L, Tan G, Wang K, Liu L, Liu J, Chen H (2015). MicroRNA-429 suppresses cell proliferation, epithelial-mesenchymal transition, and metastasis by direct targeting of BMI1 and E2F3 in renal cell carcinoma. Urol Oncol.

[70] Calura E, Fruscio R, Paracchini L, Bignotti E, Ravaggi A, Martini P, Sales G, Beltrame L, Clivio L, Ceppi L, Di Marino M, Fuso Nerini I, Zanotti L (2013). MiRNA landscape in stage I epithelial ovarian cancer defines the histotype specificities. Clin Cancer Res.

[71] Katoh M (2014). Cardio-miRNAs and onco-miRNAs: circulating miRNA-based diagnostics for non-cancerous and cancerous diseases. Front Cell Dev Biol.

[72] Lujambio A, Calin GA, Villanueva A, Ropero S, Sánchez-Céspedes M, Blanco D, Montuenga LM, Rossi S, Nicoloso MS, Faller WJ, Gallagher WM, Eccles SA, Croce CM, Esteller M (2008). A microRNA DNA methylation signature for human cancer metastasis. Proc Natl Acad Sci USA.

[73] Miles GD, Seiler M, Rodriguez L, Rajagopal G, Bhanot G (2012). Identifying microRNA/mRNA dysregulations in ovarian cancer. BMC Res Notes.

[74] Thomas M, Lange-Grünweller K, Hartmann D, Golde L, Schlereth J, Streng D, Aigner A, Grünweller A, Hartmann RK (2013). Analysis of transcriptional regulation of the human miR-17-92 cluster; evidence for involvement of Pim-1. Int J Mol Sci.

[75] Zhang W, Wang YE, Zhang Y, Leleu X, Reagan M, Zhang Y, Mishima Y, Glavey S, Manier S, Sacco A, Jiang B, Roccaro AM, Ghobrial IM (2014). Global epigenetic regulation of microRNAs in multiple myeloma. PLoS One.

[76] Feng B, Wang R, Song HZ, Chen LB (2012). MicroRNA-200b reverses chemoresistance of docetaxel-resistant human lung adenocarcinoma cells by targeting E2F3. Cancer.

[77] Rui W, Bing F, Hai-Zhu S, Wei D, Long-Bang C (2010). Identification of microRNA profiles in docetaxel-resistant human non-small cell lung carcinoma cells (SPC-A1). J Cell Mol Med.

[78] Gao Y, Chen L, Song H, Chen Y, Wang R, Feng B (2016). A double-negative feedback loop between E2F3b and miR- 200b regulates docetaxel chemosensitivity of human lung adenocarcinoma cells. Oncotarget.

[79] Peng C, Li N, Ng YK, Zhang J, Meier F, Theis FJ, Merkenschlager M, Chen W, Wurst W, Prakash N (2012). A unilateral negative feedback loop between miR-200 microRNAs and Sox2/E2F3 controls neural progenitor cell-cycle exit and differentiation. J Neurosci.

[80] Emmrich S, Pützer BM (2010). Checks and balances: E2F-microRNA crosstalk in cancer control. Cell Cycle.

[81] Schulte JH, Horn S, Otto T, Samans B, Heukamp LC, Eilers UC, Krause M, Astrahantseff K, Klein-Hitpass L, Buettner R, Schramm A, Christiansen H, Eilers M (2008). MYCN regulates oncogenic MicroRNAs in neuroblastoma. Int J Cancer.

[82] Strieder V, Lutz W (2003). E2F proteins regulate MYCN expression in neuroblastomas. J Biol Chem.

[83] Yang Y, Chang S, Zhao Z, Hou NI, He K, Wang X, Gao L, Wang L, Cai D, Guo BO, Tong D, Song T, Huang C (2015). MicroRNA-214 suppresses the proliferation of human hepatocellular carcinoma cells by targeting E2F3. Oncol Lett.

[84] Leone G, DeGregori J, Yan Z, Jakoi L, Ishida S, Williams RS, Nevins JR (1998). E2F3 activity is regulated during the cell cycle and is required for the induction of S phase. Genes Dev.

[85] Oeggerli M, Tomovska S, Schraml P, Calvano-Forte D, Schafroth S, Simon R, Gasser T, Mihatsch MJ, Sauter G (2004). E2F3 amplification and overexpression is associated with invasive tumor growth and rapid tumor cell proliferation in urinary bladder cancer. Oncogene.

[86] Tarasov V, Jung P, Verdoodt B, Lodygin D, Epanchintsev A, Menssen A, Meister G, Hermeking H (2007). Differential regulation of microRNAs by p53 revealed by massively parallel sequencing: miR-34a is a p53 target that induces apoptosis and G1-arrest. Cell Cycle.

[87] Woods K, Thomson JM, Hammond SM (2007). Direct regulation of an oncogenic micro-RNA cluster by E2F transcription factors. J Biol Chem.

[88] Xie SY, Li YJ, Wang PY, Jiao F, Zhang S, Zhang WJ (2010). miRNA-regulated expression of oncogenes and tumor suppressor genes in the cisplatin-inhibited growth of K562 cells. Oncol Rep.

[89] Chiang AC, Massagué J (2008). Molecular basis of metastasis. N Engl J Med.

[90] Di Leva G, Garofalo M, Croce CM (2014). MicroRNAs in cancer. Annu Rev Pathol.

[91] Khanbabaei H, Teimoori A, Mohammadi M (2016). The interplay between microRNAs and Twist1 transcription factor: a systematic review. Tumour Biol.

[92] Wang YW, Chen X, Ma R, Gao P (2016). Understanding the CREB1-miRNA feedback loop in human malignancies. Tumour Biol.

[93] Li Y, Wei Y, Guo J, Cheng Y, He W (2015). Interactional role of microRNAs and bHLH-PAS proteins in cancer (Review). Int J Oncol.

